# The impact of preoperative CT-based 3D visualization model on ureteric access sheath placement failure without pre-stenting

**DOI:** 10.1186/s12894-026-02197-z

**Published:** 2026-05-30

**Authors:** Yetong Zhang, Chengda Hu, Han Li

**Affiliations:** 1 Department of Urology, Chengdu Integrated TCM & Western Medicine Hospital(Chengdu First People’s Hospital), Chengdu, China; 2 Chengdu University of Traditional Chinese Medicine, Chengdu, China

**Keywords:** 3D visualization, FANS, RIRS

## Abstract

**Background:**

Placement failure of the flexible and navigable suction ureteral access sheath (FANS) with negative pressure suction may occur during retrograde intrarenal surgery (RIRS) due to ureteral stricture, hemorrhage, injury and other factors. This study aimed to investigate the predictive value of parameters from a 3D visualization model based on preoperative computed tomography (CT) for the failure of FANS placement without pre-stenting.

**Methods:**

We retrospectively analyzed the clinical data of 113 patients who underwent RIRS at Chengdu Hospital of Integrated Traditional Chinese and Western Medicine. A 3D visualization model was constructed based on preoperative CT data to measure the lateral ureteral angle, intramural ureteral diameter, intramural ureteral length and pelvic ureteral diameter. A predictive model for failure of FANS placement without pre-stenting was established after univariate and multivariate analyses, and the model performance was evaluated by bootstrap internal validation, calibration curves, decision curve analysis (DCA) etc.

**Results:**

Among the 113 patients, significant intergroup differences were observed between the successful placement group (*n* = 96) and the failed placement group (*n* = 17) in age (*P* = 0.010), lateral ureteral angle (*P* = 0.018), intramural ureteral diameter (*P* < 0.001) and pelvic ureteral diameter (*P* = 0.008). No significant intergroup differences were found in body mass index (BMI), stone CT value, stone size, stone number, gender, hydronephrosis, history of ipsilateral lithotripsy, preoperative fever history or intramural ureteral length (*P* > 0.05). Multivariate analysis identified intramural ureteral diameter (OR = 0.06, 95% CI: 0.01~ 0.29, *P* < 0.001) and pelvic ureteral diameter (OR = 0.26, 95% CI: 0.08 ~ 0.78, *P* = 0.017) as protective factors against placement failure, while the lateral ureteral angle (OR = 1.17, 95% CI: 1.04 ~ 1.31, *P* = 0.006) was a risk factor for placement failure. Due to sample size limitations, the lateral ureteral angle and intramural ureteral diameter were included in the predictive model based on the Bayesian information criterion (BIC). The area under the curve (AUC) of the model was 0.852 (95% CI: 0.763 ~ 0.941), and the AUC of internal validation via Bootstrap resampling (*n* = 1000) was 0.846 (95% CI: 0.808 ~ 0.860).

**Conclusions:**

The intramural ureteral diameter and lateral ureteral angle measured by the 3D visualization model based on preoperative CT are powerful predictors for the failure of FANS placement without pre-stenting. The constructed predictive model exhibits good performance. However, further validation with multi-center and large-sample studies is still required.

**Supplementary Information:**

The online version contains supplementary material available at 10.1186/s12894-026-02197-z.

## Introduction

Currently, the flexible and navigable suction ureteral access sheath (FANS) is an emerging device for RIRS. Its unique continuous negative pressure suction provides a clearer surgical field of view, a higher stone-free rate (SFR), lower intrarenal pressure and fewer postoperative complications compared with traditional ureteroscopes [1, 2]. However, the success of primary placement of the flexible ureteral access sheath is not only related to the selection of sheath size, but also directly associated with the ureteral course angle, ureteral diameter and ureteral wall thickness. A thinner sheath diameter should theoretically offer a greater advantage for passing through the ureteral lumen, crossing the ureteropelvic junction, entering the renal pelvis and reaching the target calyx. But it also reduces the perfusate flow rate, limits the passage of stone fragments and impairs the flexible deflection ability of the ureteroscope during surgery [3, 4]. Scholars such as Basri [5] have pointed out that limited diameters of the mid-ureter (at the iliac vessel level) and upper ureter are important factors leading to difficult UAS placement. Therefore, more accurate preoperative assessment of ureteral morphology can provide more valuable decision-making information for determining the feasibility of RIRS and the surgical approach.

Due to the narrow ureteral lumen and physiological peristalsis, conventional imaging has limited ability to depict detailed ureteral features. A recently developed three-dimensional (3D) reconstruction technique based on preoperative CT scans offers surgeons a stereoscopic and accurate visualization of ureteral anatomy [6], facilitating precise intraoperative measurements of key parameters such as ureteral diameter and ureteral angulation. Previous studies have demonstrated the advantages of CT 3D reconstruction in establishing percutaneous nephrolithotomy, partial nephrectomy and navigating complex renal calculi surgery [7, 8]. However, no studies have evaluated the effectiveness of 3D reconstruction technology as a preoperative and intraoperative planning tool for flexible ureteroscopy lithotripsy.

In this retrospective study, we measured the lateral ureteral angle (LUA), intramural ureteral diameter (IUD), intramural ureteral length and pelvic ureteral diameter (PUD) based on a preoperative 3D CT visualization model, combined with the perioperative and clinical data of patients with urinary calculi, to construct a predictive model for UAS placement failure and evaluate the clinical application of 3D CT reconstruction technology in preoperative planning for flexible ureteroscopy lithotripsy.

## Materials and methods

### Patient selection

We retrospectively analyzed 113 patients diagnosed with obstructive upper ureteral calculi who were treated at Chengdu Hospital of Integrated Traditional Chinese and Western Medicine from January 2021 to December 2023. Among these patients, 17 cases experienced failure of ureteral access sheath placement due to ureteral stricture. Four patients underwent sheathless flexible ureteroscopic holmium laser lithotripsy only, and 13 patients received secondary lithotripsy after indwelling a ureteral stent.

Inclusion criteria: (1) Patients with obstructive upper urinary tract calculi (diameter 0.6 cm ~ 2.5 cm) confirmed by preoperative diagnosis; (2) Complete preoperative urinary system CT images; (3) Underwent transurethral flexible ureteroscopic holmium laser lithotripsy; (4) Aged 18 to 80 years.

Our center adopts a one-stage laser lithotripsy protocol without preoperative JJ stent placement. Exclusion criteria: (1) Simultaneous bilateral renal calculi surgery; (2) History of ipsilateral ureteral reconstruction; (3) Ipsilateral ureteral stent implantation(due to uncontrolled infection, heavy stone burden, or multiple comorbidities etc.) or endoscopic surgery within three months; (4) Ipsilateral ureteral calculi and ureteral malignant tumors; (5) Pregnant patients; (6) Urinary tract malignant tumors and congenital urinary system anatomical abnormalities (e.g., horseshoe kidney, polycystic kidney); (7) Severe symptoms or dysfunction (severe hematuria, uncontrollable pain, cardiac, pulmonary, renal dysfunction).

### Patient charateristics 

Preoperative clinical baseline data were recorded, including age, gender, BMI, stone size (calculated as the maximum diameter of calculi on CT; for multiple calculi, the maximum diameter of the largest stone), stone CT value, number of calculi, preoperative fever and hydronephrosis. Imaging parameters measured via the 3D visualization CT model included the LUA (angle between the line connecting the ureteral orifice and the most lateral point of the ureter and the vertical midline), IUD (the transverse inner diameter of the proximal ureteral segment traversing the bladder wall), intramural ureteral length (length from the outer bladder wall to the mucosal orifice of the ureter) and PUD (the transverse inner diameter of the pelvic ureteral segment below the iliac vessels).

This study was approved by the Biomedical Ethics Committee of Chengdu Hospital of Integrated TCM & Western Medicine. In addition, all participants signed informed consent forms for surgery and the 3D CT reconstruction technology before the operation.

### 3D CT reconstruction

**Fig. 1:**
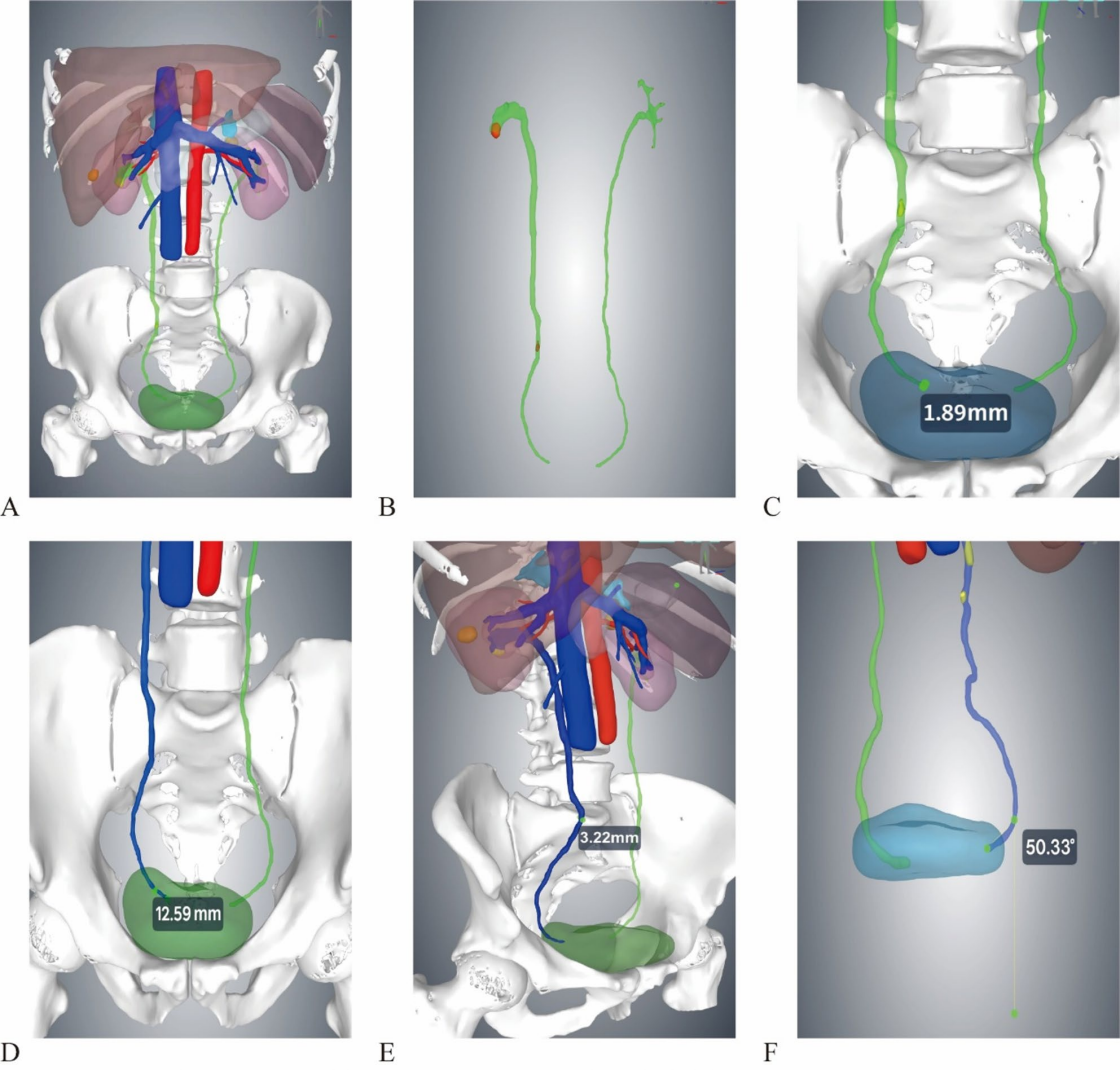
Schematic diagrams of the 3D visualization model: (**A**) Overall schematic; (**B**) Bilateral ureters and calculi; (**C**) IUD; (**D**) Intramural ureteral length; (**E**) PUD; (**F**) LUA

All patients underwent enhanced CT urography using a 64-slice CT scanner. CT images were then processed and stored in Digital Imaging and Communications in Medicine (DICOM) format. DICOM files were loaded into a 3D visualization system (IPS System, Yuke Tal, Shenzhen, China), and a 3D imaging model was constructed by professional technical engineers. The 3D CT model provided insights into the shape and location of lesions. The renal parenchyma, renal collecting system, ureter, bladder and calculi were displayed in different colors (Fig. 1). Adjusting the transparency and rotation angle of the model allowed visualization of the location and diameter of ureteral strictures, the ureterovesical angle, urinary calculi and the 3D anatomical relationship of different structural combinations. Length and angle measurement tools were used to assess stone size, ureteral diameter and the ureterovesical angle.

Measurement reproducibility was evaluated using intraclass correlation coefficient (ICC) in the first 50 patients to enhance the stability and reliability of parameter measurements. Urologist A completed all measurements and re‑measured the first 50 patients at a separate time point. Urologist B independently measured the same 50 cases in a blinded fashion.

### Surgical procedure

All surgical procedures were co-operated by two experienced urologists. Patients underwent general anesthesia in the lithotomy position. A hydrophilic guidewire (Cook Medical, BWS-035150) was advanced into the upper ureter under direct ureteroscopic vision.

FANS placement failure was defined as failure to successfully insert either a 12/14 Fr or a 10/12 Fr FANS. If placement of the FANS was not feasible due to ureteral narrowing, an attempt will be made to place a smaller 10/12 Fr FANS. If successful placement of the 10/12 Fr FANS was still not achievable, a double-J stent will be inserted, and staged ureteroscopic lithotripsy will be performed two weeks later. An irrigation pressure pump was used with an irrigation flow rate of 50–150 mL/min and suction pressure controlled at 80–120 mmHg.

Under flexible ureteroscopic guidance, the sheath was positioned adjacent to the target calyx and calculi. Lithotripsy was performed using a 200-µm holmium laser fiber (Olympus Medical) with energy settings of 0.6–2.0 J and a frequency of 10–20 Hz. The choice between pulverization and fragmentation techniques was determined by stone size (pulverization for stones < 10 mm, fragmentation for stones ≥ 10 mm) and intraoperative assessment of stone hardness. The FANS(Welllead Medical) was used to aspirate the stones from each calyx after lithotripsy. Following complete clearance of calculi from the renal pelvis and calyces, the integrity of the renal pelvic mucosa was confirmed with no significant hemorrhage observed. A double-J stent (Cook Medical) was routinely placed postoperatively at the end of the procedure.

## Statistical analysis

Statistical analyses were performed using SPSS 24.0 (IBM Corp., Armonk, NY, USA) and R (version 4.1.2). Continuous variables were expressed as mean ± SD (normally distributed) or median (IQR) (non-normally distributed). Categorical variables were reported as counts (%) and analyzed using χ² or Fisher’s exact test. Differences between groups were assessed by independent t-tests (normal) or Mann-Whitney U tests (non-normal). *P* < 0.05 (two-tailed) was considered significant.

Univariate and multivariate logistic regression identified predictors, with Bayesian Information Criterion (BIC) guiding model parsimony. A predictive model was established and internally validated via bootstrap (1,000 resamples). Performance was evaluated using the Hosmer-Lemeshow test, calibration curves, and decision curve analysis (DCA).

## Results

### Patients characteristics

The clinical data of 113 patients included age, BMI, stone size, stone number, stone CT value, hydronephrosis and history of ipsilateral renal calculi surgery; the 3D visualization model data included the LUA, IUD, intramural ureteral length and PUD. A total of 113 patients were enrolled, with 17 cases of sheath placement failure and 96 cases of successful placement, resulting in a success rate of 84.9%. Twenty patients received a thinner access sheath (10/12 Fr), and 76 patients received a wider access sheath (12/14 Fr).

The overall intra-observer ICC was 0.823 (range 0.793–0.847), and the overall inter-observer ICC was 0.807(range0.776-0.846), confirming good reproducibility and reliability of the 3D CT reconstruction–based measurements (Supplementary Table 1). Among the 113 participants, the patients in the failed placement group (*n* = 17) were significantly younger than those in the successful placement group (*n* = 96) (42.12 ± 13.95 years vs. 50.98 ± 12.60 years; *P* = 0.010). The LUA was significantly larger in the successful placement group (52.19 ± 8.47° vs. 47.68 ± 6.88°; *P* = 0.018), while the IUD (1.53[1.37–2.24]mm vs. 2.41[2.08–2.77]mm; *P* < 0.001) and PUD (3.28[2.62–3.58]mm vs. 3.88[3.25–4.21]mm; *P* = 0.008) were significantly smaller in the failed placement group. No significant intergroup differences were observed in BMI, stone CT value, stone size, stone number, gender, hydronephrosis, history of ipsilateral lithotripsy or preoperative fever (all *P* > 0.05) (Table 1).

**Table 1:** Clinical baseline characteristics of enrolled patients

Variable	Total Sample(*n* = 113)	Successful Placement(*n* = 96)	Failed Placement(*n* = 17)	Statistic	*P* Value
Age, Mean ± SD	49.65 ± 13.13	50.98 ± 12.60	42.12 ± 13.95	t = 2.63	0.010
Gender, n(%)				χ²=0.44	0.508
Female	45 (39.82)	37 (38.54)	8 (47.06)		
Male	68 (60.18)	59 (61.46)	9 (52.94)		
BMI, Mean ± SD	24.52 ± 3.05	24.48 ± 2.83	24.80 ± 4.16	t=-0.31	0.761
Number of Stones, M (Q₁, Q₃)	1.00 (1.00, 1.00)	1.00 (1.00, 1.00)	1.00 (1.00, 1.00)	Z=-0.47	0.636
Stone Size(cm), M (Q₁, Q₃)	1.20 (1.00, 1.60)	1.30 (1.00, 1.70)	1.10 (0.90, 1.40)	Z=-1.30	0.195
Stone CT Value, Mean ± SD	1278.19 ± 303.00	1263.46 ± 313.13	1361.41 ± 227.67	t=-1.23	0.221
Hydronephrosis, n (%)				-	0.618
None	7 (6.19)	5 (5.21)	2 (11.76)		
Mild	79 (69.91)	67 (69.79)	12 (70.59)		
Moderate	21 (18.58)	18 (18.75)	3 (17.65)		
Severe	6 (5.31)	6 (6.25)	0 (0.00)		
History of Ipsilateral Lithotripsy, n(%)				χ²=2.49	0.114
None	106 (93.81)	92 (95.83)	14 (82.35)		
Yes	7 (6.19)	4 (4.17)	3 (17.65)		
Preoperative Fever, n(%)				χ²=0.00	1.000
None	105 (92.92)	89 (92.71)	16 (94.12)		
Yes	8 (7.08)	7 (7.29)	1 (5.88)		
LUA, Mean ± SD	48.36 ± 7.28	47.68 ± 6.88	52.19 ± 8.47	t=-2.40	0.018
Intramural Ureteral Length (cm), Mean ± SD	1.47 ± 0.23	1.46 ± 0.23	1.50 ± 0.25	t=-0.68	0.499
IUD, M (Q₁, Q₃)	2.34 (2.02, 2.74)	2.41 (2.08, 2.77)	1.53 (1.37, 2.24)	Z=-3.85	< 0.001
PUD (mm), M (Q₁, Q₃)	3.70 (3.21, 4.18)	3.88 (3.25, 4.21)	3.28 (2.62, 3.58)	Z=-2.67	0.008

Data are presented as mean ± standard deviation (SD), median (interquartile range, IQR) or number (percentage, %)

*LUA* Lateral ureteral angle, *IUD* Intramural ureteral diameter, *PUD* Pelvic ureteral diameter

### Results of univariate and multivariate analyses

Univariate analysis showed that four indicators were statistically associated with the risk of placement failure: age (OR = 0.95, 95%CI: 0.91 ~ 0.99, *P* = 0.013), LUA (OR = 1.10, 95%CI: 1.01 ~ 1.19, *P* = 0.022), IUD (OR = 0.09, 95%CI: 0.03 ~ 0.32, *P* < 0.001) and PUD (OR = 0.30, 95%CI: 0.13 ~ 0.70, *P* = 0.008) (Table 2). To further identify independent predictors, we included indicators with *P* < 0.05 from the univariate analysis into the multivariate model to control for confounding effects. Multivariate analysis confirmed that IUD (OR = 0.06, 95% CI: 0.01 ~ 0.29, *P* < 0.001) and PUD (OR = 0.26, 95% CI: 0.08 ~ 0.78, *P* = 0.017) were protective factors against placement failure, and the LUA (OR = 1.17, 95% CI: 1.04 ~ 1.31, *P* = 0.006) was a risk factor for placement failure (Table 3).

**Table 2:** Univariate analysis for predicting ureteral access sheath placement failure

Variables	β	SE	Z	*P* Value	95%CI
Gender					
female					1.00(Reference)
male	-0.35	0.53	-0.66	0.510	0.71(0.25 ~ 1.99)
Hydronephrosis					
None					1.00(Reference)
Mild	-0.80	0.89	-0.90	0.368	0.45(0.08 ~ 2.58)
Moderate	-0.88	1.04	-0.84	0.401	0.42(0.05 ~ 3.22)
Severe	-16.65	1615.10	-0.01	0.992	0.00(0.00~∞)
History of Ipsilateral Lithotripsy					
None					1.00(Reference)
Yes	1.60	0.82	1.96	0.051	4.93(1.00 ~ 24.39)
Preoperative Fever					
None					1.00(Reference)
Yes	-0.23	1.10	-0.21	0.835	0.79(0.09 ~ 6.90)
Number of Stones	-0.56	0.97	-0.57	0.565	0.57(0.09 ~ 3.81)
Age	-0.06	0.02	-2.48	0.013	0.95(0.91 ~ 0.99)
BMI	0.04	0.09	0.41	0.685	1.04(0.87 ~ 1.23)
Stone CT Value	0.00	0.00	1.22	0.221	1.00(1.00 ~ 1.00)
Stone Size	-0.86	0.63	-1.38	0.168	0.42(0.12 ~ 1.44)
LUA (°)	0.09	0.04	2.29	0.022	1.10(1.01 ~ 1.19)
IUD(mm)	-2.39	0.63	-3.79	< 0.001	0.09(0.03 ~ 0.32)
Intramural Ureteral Length (cm)	0.76	1.12	0.68	0.495	2.15(0.24 ~ 19.32)
PUD (mm)	-1.19	0.43	-2.78	0.005	0.30(0.13 ~ 0.70)

*β* Regression coefficient, *SE* Standard error, *Z* Z value, *LUA* Lateral ureteral angle, *IUD* Intramural ureteral diameter, *PUD* Pelvic ureteral diameter, *CI* Confidence interval

**Table 3:** Multivariate analysis for predicting ureteral access sheath placement failure

Variables	β	SE	Z	*P* Value	*95%CI*
Age	-0.02	0.03	-0.87	0.386	0.98(0.92 ~ 1.03)
LUA (°)	0.16	0.06	2.73	0.006	1.17(1.04 ~ 1.31)
IUD (mm)	-2.75	0.78	-3.54	< 0.001	0.06(0.01 ~ 0.29)
PUD (mm)	-1.36	0.57	-2.39	0.017	0.26(0.08 ~ 0.78)

*β* Regression coefficient, *SE* Standard error, *Z* Z value, *LUA* Lateral ureteral angle, *IUD* Intramural ureteral diameter, *PUD* Pelvic ureteral diameter, *CI* Confidence interval

### Predictive model for sheath placement failure

Given the relatively small sample size and low incidence of placement failure in this study, we applied the 10 events per variable (EPV) criterion [9] to prevent overfitting in the predictive model. Three candidate predictors LUA, IUD, and PUD were evaluated. Using a Bayesian Information Criterion (BIC) selection principle, we constructed binary logistic regression models for all pairwise combinations of these variables and calculated their BIC values. The results demonstrated that the LUA + IUD combination yielded the lowest BIC value (81.46), which was significantly lower than those of the LUA + PUD (95.19) and IUD + PUD (84.25) (Supplementary Table 2).

Finally, the LUA and IUD were selected for inclusion in the predictive model. The model exhibited an AUC of 0.852 (95%CI: 0.763–0.941), an accuracy of 0.894 (95%CI: 0.822–0.944), a sensitivity of 0.647 (95%CI: 0.420–0.874) and a specificity of 0.938 (95%CI: 0.889–0.986) (Supplementary Table 3). The Hosmer-Lemeshow test indicated good model fit (*P* = 0.449). Due to the small sample size of this preliminary exploratory study, bootstrap resampling was further used for internal validation to evaluate model performance. After 1000 resampling, the AUC of internal validation was 0.846 (95%CI: 0.808–0.860), indicating stable model performance within the present cohort. The logistic regression equation for the model was established as follows:

$$\mathrm{Logit}(\mathrm P)\;=\;\ln(\mathrm P/(1-\mathrm P))\;=\;-2.99637\;+\;0.14247\;\times\;\mathrm{LUA}\;-\;2.81876\times\mathrm{IUD}$$

**Fig. 2:**
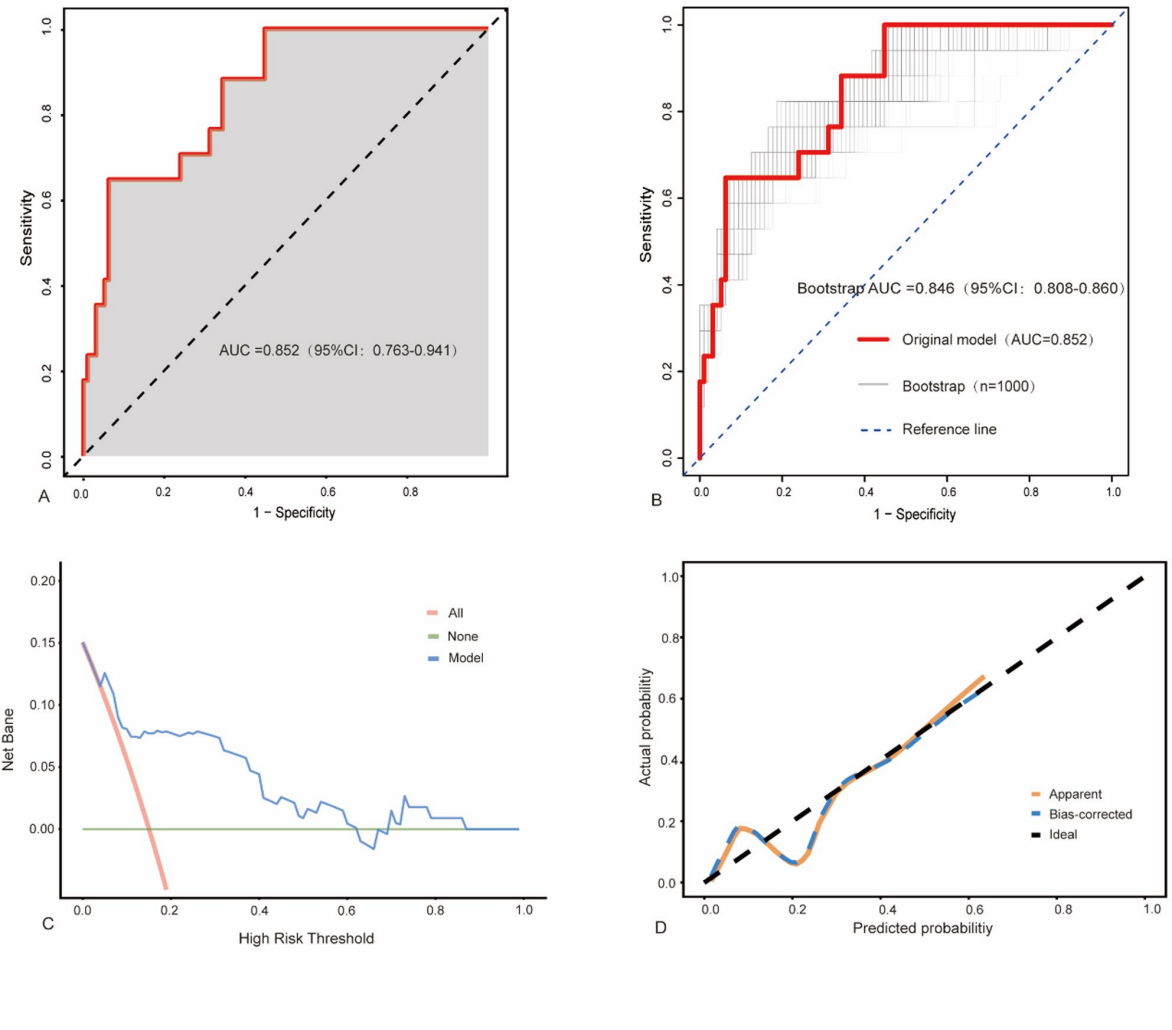
Predictive model for ureteral access sheath placement failure: (**A**) ROC curve; (**B**) Bootstrap (*n* = 1000) internal validation ROC curve; (**C**) DCA curve; (**D**) Calibration curve

**Fig. 3:**
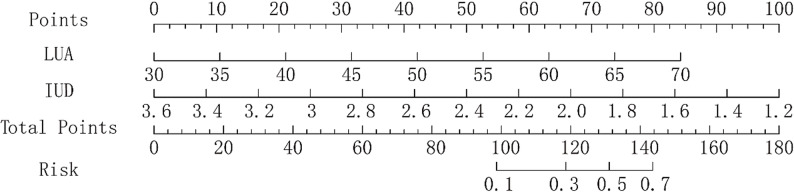
Nomogram of the predictive model for ureteral access sheath placement failure

However, internal validation based on bootstrap resampling only reflects model reproducibility within the current dataset and does not represent external validation in an independent population. Given the limited sample size, the present model is still preliminary in nature, and its generalization ability needs to be further confirmed by external validation with larger samples. The ROC curve, calibration curve and DCA curve are shown in Fig. 2, and the nomogram of the predictive model is shown in Fig. 3.

## Discussion

The primary surgical endpoint for urinary calculi is achieving a stone-free state. The introduction of the novel FANS in RIRS has significantly improved the stone-free rate after lithotripsy and reduced the overall incidence of postoperative infectious complications [10]. Currently, some medical centers adopt a two-stage approach: primary indwelling of a ureteral stent followed by secondary laser lithotripsy two weeks later. Preoperative stenting enhances the success rate of secondary sheath placement and minimizes intraoperative ureteral injury [11, 12]. However, with the launch of thinner negative-pressure suction ureteral access sheath and against the backdrop of the current economic downturn, more refined multidimensional assessment of patients’ ureteral morphology enables the selection of appropriate candidates for primary laser lithotripsy. This approach conserves medical resources and reduces patients’ economic burden without compromising medical quality.

We constructed a prediction model using logistic regression based on two preoperative anatomical indicators: LUA and IUD. The former reflects ureteral tortuosity, and the latter defines the narrowest channel for sheath insertion. With a cutoff value of 0.35, the model enables effective risk stratification: a predicted probability ≥ 0.35 suggests a high risk of difficult sheath placement, indicating the preoperative stenting; a probability < 0.35 indicates low risk, allowing routine use of standard sheaths. This concise and objective model offers clear guidance for individualized clinical decision-making and optimizes perioperative management.

Previous studies on predictive models for FANS placement failure have identified various risk factors. Luo et al. reported that age (OR = 0.95, *P* < 0.001), male gender (OR = 2.15, *P* = 0.017), body mass index (BMI) (OR = 1.12, *P* < 0.001), history of stone expulsion (OR = 0.35, *P* = 0.014), and ureteral stone diameter (OR = 0.23, *P* < 0.001) were significant independent risk factors, with an area under the receiver operating characteristic curve (AUC) of 0.789 [13]. In contrast, Sene et al. identified female gender, a history of urinary tract infection, and younger age as risk factors for failure of 9.5-11.5Fr sheath placement [14]. Focusing on ureteral morphological parameters, Azhar et al. found that a tent-shaped ureteral orifice after guidewire placement was associated with a higher success rate of primary FANS placement [15]. The ureter is not a regular circular lumen, and the degree of physiological folding and torsion of the ureteral mucosa is also an important factor affecting sheath placement [16]. When ureteral structures are quantified via preoperative imaging parameters, Cho et al. reported in their CT-based predictive model that the distal ureteral lateralization angle (the angle between the bladder orifice and the lateralmost point of the distal ureter) was an effective predictor of sheath placement failure [17, 18].

In our study, age and pelvic ureteral diameter also exhibited predictive value in univariate analysis but were excluded from the final model due to sample size limitations to avoid overfitting. We established a predictive model for FANS placement failure by retrospectively analyzing ureteral parameters from a 3D visualization model based on preoperative CT scans. The main measured indicators included the lateral ureteral angle, intramural ureteral length, intramural ureteral diameter, and pelvic ureteral diameter. Through univariate and multivariate analyses, the lateral ureteral angle and intramural ureteral diameter were selected for inclusion in the model. Notably, the model was validated internally using bootstrap resampling, which does not equate to external validation. Given the relatively small sample size and limited number of positive events in this study, the reported performance metrics should be interpreted with caution, as they may be somewhat optimistic. These results should be regarded as preliminary, and further external validation in larger and independent cohorts is warranted to verify the generalizability and clinical applicability of the model.Currently, there remains no consensus on the personalized selection of FANS sizes in clinical practice. Compared with the 10/12Fr sheath, the 11/13Fr FANS exhibits an approximately threefold higher suction rate [19]. Inappropriate selection of sheath size may compromise stone clearance efficiency and elevate the risk of postoperative ureteral stricture. A 9.2Fr endoscope is anticipated to induce higher intraoperative intrarenal pressure than a 7.5Fr endoscope. A larger working channel can effectively reduce the time to restore initial pressure after negative-pressure suction and allow the passage of larger stone fragments [20]. Tracy et al. found that using a 14/16Fr UAS compared with a 12/14Fr UAS achieved a similar stone-free rate and higher surgical efficiency without increasing the risk of postoperative complications or ureteral injury [21]. However, Aykanat et al. reported on the contrary that using a 12/14Fr sheath compared with a 9.5/11.5Fr sheath was associated with an increased risk of high-grade ureteral injury, although the difference was not statistically significant [22]. A follow-up study by Stern et al. found that preoperative hydronephrosis, shorter duration of ureteral stenting, and advanced age were risk factors for ureteral stricture after ureteral injury in RIRS [23]. Preoperative ureteral morphology, male sex, large stone size, difficulty in sheath placement and longer insertion time are all closely associated with post-retrograde intrarenal surgery (RIRS) ureteral injury [24]. Additionally, the proximal ureteral diameter is associated with high-grade ureteral injury, with a smaller diameter correlating with an increased risk. Preoperative measurement of the proximal ureteral diameter can guide FANS size selection and predict the risk of ureteral injury [25]. As an important factor closely associated with the success of FANS insertion, FANS size was not included in the predictive model analysis. This omission may lead to FANS size acting as a potential confounder, which could exert a certain impact on the interpretation of the study results and the reliability of the predicted associations between preoperative ureteral anatomical parameters and FANS placement failure.

In subsequent studies, we will extend the application of the 3D visualization model to predict postoperative complications and ureteral injury, providing more refined preoperative guidance for surgical approaches and sheath size selection.

Our model has several advantages. First, this is the first study to construct a predictive model for FANS placement failure using parameters measured from a 3D CT visualization model. Second, the parameters of the 3D visualization model are easy to measure, allowing clinicians to assess preoperative ureteral parameters using mobile devices. Third, the model includes only two parameters, reducing prediction costs. Additionally, the preoperative visualization model enables a more intuitive assessment of patients’ preoperative ureteral conditions.

However, this study has several limitations. Due to the initial promotion of flexible negative-pressure suction sheaths and 3D visualization models, this is a small-sample study with a low proportion of positive events (only 17 cases of FANS placement failure), which may limit the predictive accuracy of the model. Second, this is a single-center study lacking formal external validation; we are currently conducting external experiments to further evaluate the actual predictive value of the visualization model parameters. Third, as a retrospective study, there is a certain degree of selection bias.

## Conclusion

Measurements of the diameter of the intramural ureter and the lateral ureteral angle from preoperative CT-based 3D visualization models are effective predictors of unprepared access sheath placement failure. The developed prediction model exhibits favorable performance; however, validation in multicenter, large-sample cohorts remains necessary.

## Supplementary Information

Supplementary Material 1.

Supplementary Material 2.

Supplementary Material 3.

## Data Availability

The data supporting the findings of this study are available from the corresponding author upon reasonable request, with restrictions to protect patient privacy.
